# Lower‐risk myelodysplastic syndromes: Current treatment options for anemia

**DOI:** 10.1002/jha2.523

**Published:** 2022-08-12

**Authors:** Mathieu Meunier, Sophie Park

**Affiliations:** ^1^ Department of Haematology CHU Grenoble Alpes Grenoble France; ^2^ Institute for Advanced Bioscience Université Grenoble Alpes Grenoble France

**Keywords:** anemia, hematology, MDS, therapy

## Abstract

Myelodysplastic syndromes (MDS) are a heterogeneous group of clonal hematological disorders. Treatment options are classified and defined by prognostic risk based on the International Prognostic Scoring System (IPSS) and, more recently, the revised IPSS (IPSS‐R). The treatment goal for lower‐risk MDS is to correct cytopenias or their consequences, with the goal of maintaining or improving quality of life. Erythropoiesis‐stimulating agents (ESAs) play an important role in treating anemia. Individuals with MDS who have a 5q deletion are particularly sensitive to treatment with lenalidomide; however, this comprises the minority of patients with MDS. Luspatercept was recently approved in the United States and the European Union for the treatment of ESA‐refractory MDS with ring sideroblasts. Research into new treatment options, especially after ESA failure, is needed. In this review, we will focus on the current therapeutic options for MDS‐related anemia.

## 1 INTRODUCTION

Myelodysplastic syndromes (MDS) are a heterogeneous group of clonal diseases of the hematopoietic stem cell [1]. MDS occurs mainly in older patients and are characterized by ineffective hematopoiesis leading to cytopenia, contrasting with bone marrow hypercellularity. Disease evolution leads to the emergence of mutant genetically unstable clones and transformation to acute myeloid leukemia (AML) in about 30% of cases [2]. There are several possible goals in the treatment of MDS: correcting cytopenias, especially anemia, or alleviating their consequences; delaying the transformation to AML; prolonging survival; and improving quality of life. Which of these goals is prioritized depends on the assessment of prognostic risk as well as the patient's age and comorbidities. Therapeutic indications are based on the International Prognostic Scoring System (IPSS) [3] and, more recently, the revised IPSS (IPSS‐R) [4], which scores patients from Very low‐to Very high‐risk. In lower‐risk forms, supportive treatment is the priority. Regardless of the risk level, supportive treatment is a major component of MDS management.

The main challenge in patients with lower‐risk MDS is usually the treatment of cytopenias, as the risk of transformation to AML is relatively low. In addition, given the high median age of these patients, treatments should have low toxicity and improve patient quality of life. Anemia is the most common and usually most symptomatic cytopenia, occurring in over 80% of patients with lower‐risk MDS. Management of anemia involves repeated red blood cell (RBC) transfusions. Clinical practice, however, is increasingly using erythropoiesis‐stimulating agents (ESAs) to treat anemia. In this review, we will discuss current therapeutic options for MDS‐related anemia, as well as available treatments following ESA failure.

## 2 LONG‐TERM TRANSFUSION THERAPY FOR ANEMIA IN LOWER‐RISK MDS

Patients often receive RBC transfusions, sometimes for years, primarily following the failure of other treatments for anemia, such as ESAs. There are, however, several drawbacks associated with RBC transfusions. Throughout most of the course of the disease, patients with MDS have a hemoglobin level of less than 10 g/dL, which leads to a decreased quality of life [5] as well as a gradual deterioration of myocardial function [6]. RBC transfusion involves fairly onerous logistics, including the collection of blood products, immunohematological tests for the donor and recipient, and securing availability in an inpatient hospital or transfusion center. Although the risk of infection from transfusions is now quite low, it is still not zero. Hemolysis accidents have also not been eliminated, and transfusion‐associated circulatory overload in older patients may also occur. For these reasons, there is an increasing focus on trying to correct anemia in lower‐risk MDS through the use of drugs to improve RBC production.

Moreover, the repeated intake of iron through transfusions necessitates iron chelation therapy because of oversaturation of transferrin, resulting in nontransferrin‐bound iron and labile plasma iron (LPI). LPI can cause oxidative stress via the Fenton reaction leading to tissue dysfunction, notably in the heart, liver, and pancreas. The putative consequences of iron overload due to repeat transfusions are particularly common in patients with hemoglobinopathies. A retrospective study has shown that iron chelation therapy appears to improve survival in heavily transfused patients with lower‐risk MDS [7]. Median overall survival was 53 months in chelated patients versus 124 months in those nonchelated (*p* < 0.0003). Recently, a double‐blind, placebo‐controlled trial (TELESTO) [8] has shown improvement in event‐free survival in chelated patients. In addition, iron overload may induce genomic instability through DNA damage and promote disease progression [9]. The most commonly used iron‐chelating drug is deferasirox, which is associated with a favorable safety and efficiency profile [10]. Most MDS cooperative groups support the assessment of ferritin level as a method to monitor chelation approach, despite its association with inflammation and other comorbidities. The use of a ferritin threshold to determine the onset of iron chelation, typically set at around 1,000 ng/mL, is still, however, under debate.

## 3 ERYTHROPOIESIS‐STIMULATING AGENTS

### 3.1 ESA: Primary option for patients with symptomatic anemia

Two preliminary remarks should be made about ESA therapy: ESAs do not appear to affect the progression of the disease, nor do they alter the risk of progression to AML. However, two studies have shown that ESAs provide a survival benefit in patients with lower‐risk MDS, particularly in responders [11, 12]. When the Groupe Francophone des Myélodysplasies (GFM) series was compared historically to the series used to develop the IPSS score, the 5‐year survival of ESA‐treated patients with MDS was 64%, versus only 39% in the historical series, matched on key prognostic factors like age, French‐American‐British (FAB) classification, percentage of blasts, and karyotype. Based on the findings of a randomized trial of ESAs versus supportive treatment, a survival benefit was only confirmed in responding patients [13].

ESAs, notably epoetin alfa, have been granted marketing authorization for the indication of anemia in lower‐risk MDS by the European Medicines Agency (EMA) in Europe. The results of two large, multicenter, randomized, double‐blind, placebo‐controlled clinical trials evaluating erythropoietin (EPO) or darbepoetin versus placebo in low‐risk MDS (EPOANE3021 [NCT01381809] and ARCADE [NCT01362140]) have been finalized and published [14, 15]. In the EPOANE study, hematologic improvement – erythroid (HI‐E) response with epoetin alfa was achieved in 31.8% of patients receiving epoetin alfa versus 4.4% of patients receiving placebo. In the ARCADE study at week 24, 14.7% of patients receiving darbepoetin achieved HI‐E versus 0% for patients receiving placebo, with RBC transfusion dependence reduced from 59.2% to 26.1% with darbepoetin.

Epoetin alfa and beta biosimilars are fully bioequivalent. A study of a biosimilar of epoetin alfa in patients with lower‐risk MDS with anemia showed an International Working Group (IWG) 2006 response rate of 48%, similar to those obtained with “originator” epoetin alfa [16]. Numerous trials of epoetin alfa and epoetin beta at a dose range of 10,000 to 20,000 units three times a week have resulted in response rates of 30%–60% in patients with lower‐risk MDS. Response definitions of these trials included increases in hemoglobin levels and achievement of RBC transfusion independence; response rates were also dependent on prognostic factors. Weekly administration of EPO (60,000 IU/week) resulted in similar response rates. In addition, reducing the number of injections was found to improve the quality of life of these patients [17, 18]. Darbepoetin alfa, a glycosylated derivative of recombinant EPO, has a more prolonged action on cells of the erythroblastic lineage in MDS [19]. In a French study in which darbepoetin was administered at a dose of 300 μg once a week, the response rate was 63% [19, 20]. Other studies have reported similar results [21]. The vast majority of responses to ESAs are observed within the first 12 weeks, although some responses are observed later. The median duration of response is approximately 2 years. In contrast, the two prospective randomized trials with epoetin alfa and darbepoetin [14, 15] report erythroid response rates in the ranges of only 15%–30%, likely related to the study design of each trial. These study design limitations include underdosing of darbepoetin in the ARCADE trial, with discontinuation of the ESA as soon as the hemoglobin level reached 12 g/dL (preventing a robust and prolonged evaluation of erythroid responses), and the use of the IWG 2006 response assessment criteria, which remain imprecise and insufficient in evaluating the duration of response. On this matter, new erythroid response criteria have been proposed for the IWG 2018 response criteria, which extend HI‐E response and transfusion‐dependence assessment times to 16–24 weeks instead of the original 8 weeks [22].

### 3.2 Indication for ESA therapy

Patients who have not previously received RBC transfusions typically respond better to ESAs than those previously transfused. In the GFM series [12], the ESA response rate (per IWG 2006 criteria) [23] in nontransfused patients was 66% versus only 37% in transfused patients. The findings of a retrospective study suggest that the earlier ESA therapy is initiated following diagnosis (ideally within 6 months), the greater the probability of response and the longer the time to RBC transfusion dependence. The response rate was 76% when patients received ESA therapy within 6 months of diagnosis compared with 46% in patients receiving therapy after 6 months [24]. A prospective trial is currently underway to evaluate whether early introduction of ESA treatment delays transfusion dependence (EudraCT no.: 2016‐000327‐10, ClinicalTrials.gov Identifier: NCT03223961).

### 3.3 Prognostic factors of EPO response

The main predictors of response to EPO with or without granulocyte colony‐stimulating factor (G‐CSF) are the extent of transfusion requirements (greater or less than 2 units of packed RBCs/month) and the level of endogenous EPO (greater or less than 500 IU/L). These two variables were used to develop a score that distinguished three groups of patients with a 74%, 23%, and 7% probability of response to EPO with or without G‐CSF, depending on whether none, one, or two of these risk factors were present, in a study that did not include only lower‐risk patients with MDS [25].

Other favorable prognostic factors for response are a blast count of less than 5%; an IPSS score of less than or equal to 1; and the absence of multilineage dysplasia. In a larger series of patients, however, we did not find the absence of multilineage dysplasia to be a factor. We found that responses occurred in 56% of MDS‐EB1 cases, and patients in this category could therefore benefit from ESA therapy in the absence of an unfavorable karyotype [26]. Patients with lower‐risk MDS with 5q deletion have a significantly lower probability of response than other low‐risk MDS cases, and a shorter duration of response [27].

New biological tools could also be used to predict response to ESAs, including analysis of p‐ERK or p‐STAT expression [28]. However, these biological tests need to be validated in multiple centers in larger series of patients and are not part of standard practice. Molecular biology studies have revealed that only the cumulative number of mutated genes has an unfavorable effect on erythroid response, but this factor disappears in multivariate analysis in the presence of endogenous EPO level [29].

More recently, the contributions of flow cytometry and an improved understanding of iron metabolism have highlighted two factors that can predict erythroid response: the RED Score and the hepcidin:ferritin ratio. The RED Score quantifies dyserythropoiesis and factors in hemoglobin level and the coefficient of variation of the erythroid markers CD36 and CD71. The higher this variation, the lower the rate of erythroid response. A low hepcidin:ferritin ratio, which indicates poor iron recirculation in the body, is also associated with lower rates of erythroid response [16].

## 4 THERAPEUTIC OPTIONS FOLLOWING LOSS OF RESPONSE TO ESAs

A variable proportion of patients treated with ESAs are resistant to ESAs from the outset (primary refractory patients), while others will eventually become resistant secondarily (relapsed patients) after an initial response time close to the median response time of 2 years. Patients who do not respond after 8–12 weeks of ESA ± G‐CSF are defined as primary refractory or resistant. In retrospective series, the proportions of patients failing ESA therapy (refractory and relapsed) were approximately 50% and 26%, respectively, with a median follow‐up of 7 years. Using a 6‐month cutoff for the relapse period, we identified a population with poorer overall survival, with a median survival of 36 months after ESA failure versus 54 months (*p* = 0.02) and a cumulative incidence of transformation to AML of 21.6% versus 9.0% at 5 years [30]. In an international series involving 1700 patients, the incidence of AML was 16% versus 8% at 5 years in ESA‐refractory patients versus persistent responders > 6 months [31].

**FIGURE 1:**
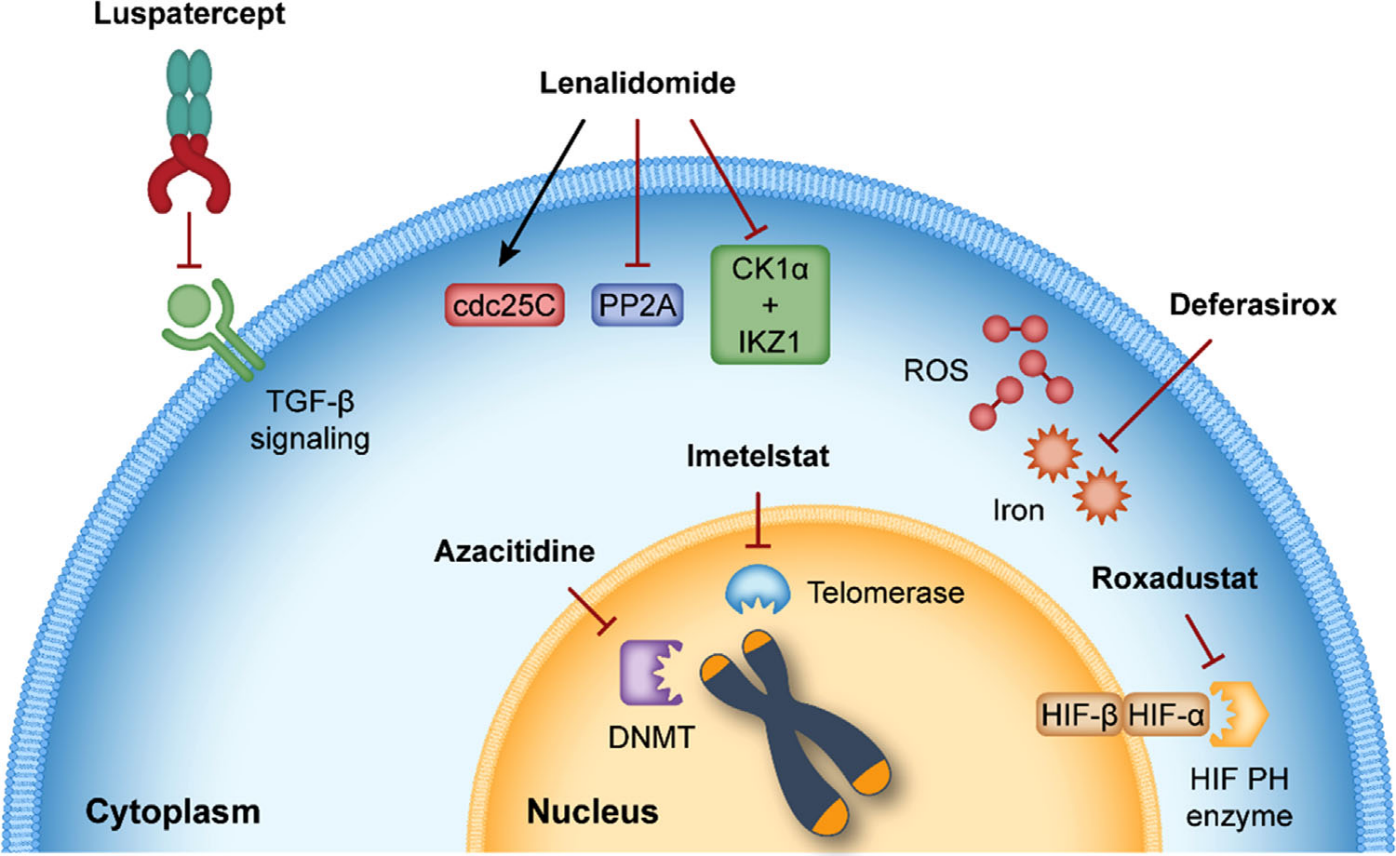
Differing mechanisms of action of treatments for anemia. cdc25C, cell division cycle 25C; CK1α, casein kinase 1 alpha; DNMT, DNA methyltransferase; HIF, hypoxia‐inducible factor; IKZ1, Ikaros family zinc finger 1; PH, prolyl hydroxylase; PP2A, protein phosphatase 2A; ROS, reactive oxygen species; TGF‐β, transforming growth factor β

**FIGURE 2:**
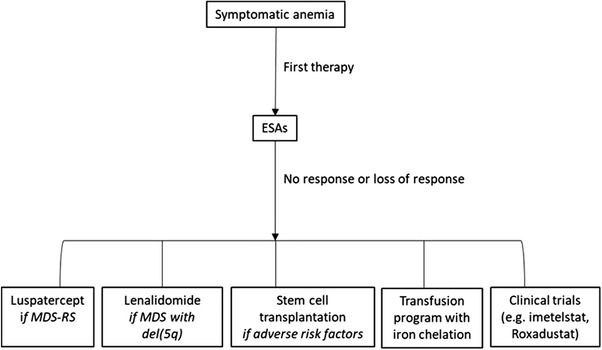
Treatment algorithm for MDS‐related anemia. del(5q), 5q deletion; ESAs, erythropoiesis‐stimulating agents; MDS, myelodysplastic syndromes; RS, ring sideroblasts

A loss of response to an ESA involves checking that the disease has not progressed to a higher stage (increased marrow blasts, appearance of cytogenetic abnormalities), which, in our experience, is confirmed in only about 30% of cases. In other patients, an additional cause of anemia (such as iron or folate deficiency) should be checked and treated. If no additional cause of anemia is found, the patient is considered ESA refractory; therapeutic options available to these patients and their different mechanisms of action are shown in Figure 1. A suggested treatment algorithm is shown in Figure 2.

### 4.1 Lenalidomide

Lenalidomide was first used to treat patients with del(5q) MDS. List et al. [32] reported 67% of 148 RBC transfusion‐dependent patients with del(5q) MDS achieved RBC transfusion independence, while 76% had a reduction of transfusion need. These results were confirmed by another clinical trial [33]. In this phase 3 trial of lenalidomide versus placebo in 205 patients with RBC transfusion‐dependent lower‐risk MDS, 56.1% of patients achieved RBC transfusion independence with 10 mg daily of lenalidomide. Lenalidomide was subsequently trialed in patients with lower‐risk, RBC transfusion‐dependent non‐del(5q) MDS who were refractory or ineligible to receive ESAs, at a dose of 10 mg/day versus placebo [34]. RBC transfusion independence with lenalidomide was achieved in 26.9% of patients versus 2.5% with placebo. A subgroup of patients with endogenous EPO levels < 100 IU/L who had received prior ESA therapy achieved RBC transfusion independence in 42.5% of cases. These two simple variables (prior ESA therapy and low endogenous EPO) could be used to select patients more likely to benefit from lenalidomide after ESA failure, as research on molecular characteristics to better identify appropriate patients continues. At present, there is no somatic mutation that predicts response to lenalidomide. Interestingly, the combination of lenalidomide with an ESA was shown to have additive effects on restoring erythropoiesis in the event of ESA failure [35]. Consistent with the results obtained with lenalidomide monotherapy, patients with EPO levels < 100 IU/L had higher rates of erythroid response. It may therefore also be beneficial to combine lenalidomide with EPO even after EPO has failed. Lenalidomide induces coalescence of lipid rafts in erythroid progenitors, leading to a concentration of EPO receptors on the surface of these cells and enhanced EPO‐induced signal transduction with downstream phosphorylation of JAK2 and STAT5 [36]. The investigators of a randomized phase 3 trial [35] proposed the use of either lenalidomide alone (10 mg/day, 21 days/4 weeks) or lenalidomide + EPO beta (60,000 U/week) in ESA‐refractory, RBC transfusion‐dependent patients with lower‐risk MDS without del(5q). The four‐cycle response was 23.1% in the lenalidomide‐only group versus 39.4% in the lenalidomide + EPO beta group (*p* = 0.04), supporting the addition of EPO to ESA‐refractory patients receiving lenalidomide. The median duration of response was 15–18 months. However, there was no difference in the rate of achieving RBC transfusion independence: 13.8% in the lenalidomide‐only group achieved transfusion independence versus 24.2% in the lenalidomide + EPO group (*p* = 0.13). Those results were confirmed by another randomized phase 3 trial which assessed lenalidomide + epoetin alfa versus lenalidomide alone [37]. The overall erythroid response rate was 46.5% for combination therapy versus 32.3% for lenalidomide monotherapy. Hematological toxicity was noteworthy, with 62% of patients reporting neutropenia and 36% reporting grades 3–4 thrombocytopenia; a dose reduction to 5 mg/day was also required in approximately 40% of patients. Lenalidomide was only considered to be beneficial for patients with favorable cytogenetics. Patients with low levels of *NPM1* expression and Cereblon A/A polymorphism have also been noted to be less likely to respond to the lenalidomide + EPO combination [38].

### 4.2 Demethylating agents

With respect to the use of demethylating agents, the GFM study randomized 5‐azacitidine administered for 5 days versus 5‐azacitidine + EPO at 60,000 IU/week [39]. Although there was a slight patient imbalance between treatment arms, with a higher proportion of patients with refractory anemia with ring sideroblasts (RARS) in the azacitidine‐only arm, the overall response rates were similar at around 16%–18%, with significant toxicity also noted, particularly related to fever and infections resulting in hospitalization. These findings therefore suggest that hypomethylating agents may not be suitable for these types of patients. Recently, a phase 3, placebo‐controlled trial evaluated CC‐486 (oral azacitidine) in patients with IPSS lower‐risk MDS and RBC transfusion‐dependent anemia with thrombocytopenia [40]. Although patients who received CC‐486 had significantly improved rates of achievement of RBC transfusion independence and durable bi‐lineage improvements, early deaths were noted to occur in the CC‐486 arm.

### 4.3 Luspatercept and other erythroid maturation agents

New therapies for MDS‐related anemia are activin A “ligand traps”, which exert antagonistic effects on activin receptors. Erythroid differentiation is mainly regulated by EPO in the early stages and inhibited by molecules of the transforming growth factor beta superfamily such as GDF11 and activin A. Activin ligand traps, which promote late erythroid differentiation, are typically referred to as erythroid maturation agents. Two types of ligand traps have been recently investigated: sotatercept (ACE‐011), which was initially intended to treat osteoporosis in postmenopausal women, and luspatercept (ACE‐536). Both molecules are administered subcutaneously every 3 weeks. Luspatercept has recently been investigated as a treatment for anemia in patients with lower‐risk MDS, including in the phase 2 PACE‐MDS study [41]. PACE‐MDS was a multicenter, open‐label, dose‐finding study of luspatercept in patients with lower‐risk MDS; results were encouraging, with 63% of patients who received higher doses of luspatercept achieving HI‐E versus 22% of those receiving lower doses. Interestingly, in the PACE‐MDS study, a higher response rate was observed in patients with MDS with ring sideroblasts (MDS‐RS) or *SF3B1* mutation. This observation was the basis for the subsequent MEDALIST trial [42]. MDS‐RS is a particular subtype of MDS characterized by a prominent erythroid dysplasia responsible for macrocytic anemia and mitochondrial iron accumulation. Patients with MDS‐RS often have systemic iron overload even before transfusion dependence [43]. *SF3B1* mutations are found in 90% of patients with MDS‐RS [44]. MEDALIST is a double‐blind, placebo‐controlled, phase 3 trial of luspatercept in patients with lower‐risk MDS‐RS, in which patients were randomly assigned to receive luspatercept or placebo subcutaneously every 3 weeks. The results of this trial led to approval by the EMA of luspatercept for adults with Very low‐ to Intermediate‐risk MDS‐RS with anemia who had failed ESA treatment and required RBC transfusion, a patient population that was lacking treatment options. A total of 229 patients were enrolled, of whom 153 received luspatercept. The MEDALIST primary end point, RBC transfusion independence of at least 8 weeks, was observed in 38% of the patients in the luspatercept arm versus 13% of those receiving placebo. Regarding hemoglobin, levels increased by roughly 1 g/L in patients treated with luspatercept, allowing them to exceed the transfusion threshold. Additionally, the study met the key secondary end point of transfusion independence of at least 12 weeks and other secondary end points including modified HI‐E. Clinical trials are ongoing in order to evaluate luspatercept in patients with lower‐risk MDS without ring sideroblasts. The aim of one clinical trial currently underway is to assess the efficacy and safety of luspatercept versus epoetin alpha in lower‐risk, ESA‐naive patients with MDS who require RBC transfusions (COMMANDS, NCT03682536). Another erythroid maturation agent, KER‐050, a modified ActRIIA ligand trap, is also being developed. A phase 2 clinical trial is currently underway to evaluate KER‐050 in patients with Very low‐ and Intermediate‐risk MDS (NCT04419649).

### 4.4 Iron chelation therapy

A better understanding of iron metabolism in erythropoiesis may lead to the possibility of providing low‐dose deferasirox to ESA‐resistant patients. A low hepcidin:ferritin ratio identifies patients who are less likely to respond to ESAs, suggesting that poor circulation of iron in the body may explain some ESA resistance [16].

Many retrospective studies have shown the effectiveness of iron chelation therapy in reducing iron burden in patients with lower‐risk MDS patients, and it appears to have positive effects on hematopoiesis in some patients with MDS, leading to reduction of RBC transfusions or even transfusion independence [45, 46, 47, 48, 49, 50, 51]. The effect of deferasirox on erythropoiesis was evaluated in vitro in lower‐risk MDS; it was found that low‐dose deferasirox protected erythroid progenitors from apoptosis by reducing levels of reactive oxygen species, leading to activation of the NF‐κB pathway to positively affect progenitor proliferation and negatively affect the inflammatory environment [52]. A clinical trial is underway to investigate low‐dose deferasirox in lower‐risk patients with MDS who are resistant or have relapsed to ESA therapy (EudraCT no.: 2017‐001258‐33, ClinicalTrials.gov Identifier: NCT03387475).

### 4.5 Imetelstat, a telomerase inhibitor

Another therapeutic target is telomerase activity. Indeed, Briatore et al. have previously demonstrated that telomerase activity and hTERT expression in bone marrow is elevated in samples from patients with MDS compared with healthy donors [53]. The telomerase inhibitor imetelstat has been evaluated in lower‐risk MDS and demonstrated encouraging response rates in a phase 2 study (MDS3001) [54]. Rates of RBC transfusion independence were 37% after 8 weeks of treatment and 23% after 24 weeks for a median duration of 86 weeks. These results are fairly similar to those reported for luspatercept. The disadvantage of this molecule, however, is that unlike luspatercept, it has cytopenic effects on other cell lines.

### 4.6 Roxadustat

Initially developed to correct anemia due to chronic kidney disease and dialysis [55, 56], roxadustat promotes erythropoiesis by increasing endogenous EPO levels. Roxadustat stabilizes hypoxia‐inducible factor (HIF) to increase the number of EPO receptors in the bone marrow and improve iron metabolism and its bioavailability. In the specific population of patients with kidney disease, roxadustat treatment resulted in a larger increase in hemoglobin level than EPO [57]. Results of MATTERHORN, a phase 3, randomized, double‐blind, placebo‐controlled study of roxadustat in patients with lower‐risk MDS and low transfusion burden have been published. Transfusion independence was achieved in 9 patients (37.5%) at 28 and 52 weeks, and ≥50% reduction in RBC transfusions was achieved in 54.2% and 58.3% of patients at 28 and 52 weeks, respectively [58].

### 4.7 Hematopoietic stem cell transplantation

The indication for allogeneic hematopoietic stem cell transplantation (HSCT) remains controversial for patients with lower‐risk MDS. For example, some patients with low‐risk IPSS scores may have aggressive MDS according to the IPSS‐R, due to the etiology of the MDS being notably therapy related, the presence of bone marrow fibrosis, genetic mutations or emergence of karyotype abnormalities, and treatment outcomes. In a retrospective analysis of 246 patients with MDS in the European Society for Blood and Marrow Transplantation database who underwent transplantation due to IPSS‐classified low or intermediate‐1 disease, 76% were reclassified as intermediate or higher risk according to the IPSS‐R. Patients with lower‐risk MDS had better outcomes than those with higher risk after HSCT, with an overall survival of 58% and progression‐free survival of 54% at 3 years post‐transplantation. In a multivariate analysis in that study, adverse risk factors for progression‐free survival were found to be marrow blast percentage, donor/recipient cytomegalovirus serostatus, and the source of the stem cells [59]. These data suggest that a prospective study is needed. The MDS‐ALLO‐RISK study (NCT02757989) was designed to answer this question; 79 patients were included, 64 of whom had a donor and 15 of whom did not. Three‐year overall survival was 60% and 64.2% in the two groups, respectively (*p* = not significant). Unfortunately, the study was stopped due to futility [60].

## 5 CONCLUSIONS

There is growing interest in treating patients with anemia who are ESA‐refractory or unresponsive to ESAs; these patients may have a poorer prognosis.

Luspatercept recently received FDA and EMA approval for MDS‐RS refractory to ESAs. Identification of additional adverse prognostic factors would help define a group of patients for whom azacitidine and HSCT may be beneficial. Other therapeutic options in addition to EPO are currently being investigated, such as telomerase inhibitors, HIF stabilizers, and iron metabolism modifiers (Figure 2).

## CONFLICT OF INTEREST

M. M. has no competing interests to declare. S. P. reports advisory board and research grants from BMS/Celgene, Novartis, Pfizer, and Takeda; research grant from Sandoz; speaker honoraria from BMS/Celgene, Novartis, and Takeda.

## FUNDING INFORMATION

The authors received no specific funding for this work.

## ETHICS STATEMENT

No institutional review board approvals were required; no informed written/verbal consent from patients or patient's parents/guardians was required. The manuscript is a review of the current published literature about the treatment options for anemia in patients with lower‐risk MDS.

## AUTHOR CONTRIBUTIONS

M. M. and S. P. performed the literature search and wrote the review. Both authors are fully responsible for all content and editorial decisions

## Data Availability

BMS policy on data sharing may be found at https://www.bms.com/researchers‐and‐partners/independent‐research/data‐sharing‐request‐process.html.
